# Thermo-Electro-Mechanical Vibrations of Porous Functionally Graded Piezoelectric Nanoshells

**DOI:** 10.3390/nano9020301

**Published:** 2019-02-20

**Authors:** Yun Fei Liu, Yan Qing Wang

**Affiliations:** 1 Department of Mechanics, College of Sciences, Northeastern University, Shenyang 110819, China; lyfboook@163.com; 2 Key Laboratory of Ministry of Education on Safe Mining of Deep Metal Mines, Northeastern University, Shenyang 110819, China

**Keywords:** functionally graded piezoelectric nanoshells, nano-void, Love’s shell theory, nonlocal elasticity theory, size effect, vibration

## Abstract

In this work, we aim to study free vibration of functionally graded piezoelectric material (FGPM) cylindrical nanoshells with nano-voids. The present model incorporates the small scale effect and thermo-electro-mechanical loading. Two types of porosity distribution, namely, even and uneven distributions, are considered. Based on Love’s shell theory and the nonlocal elasticity theory, governing equations and corresponding boundary conditions are established through Hamilton’s principle. Then, natural frequencies of FGPM nanoshells with nano-voids under different boundary conditions are analyzed by employing the Navier method and the Galerkin method. The present results are verified by the comparison with the published ones. Finally, an extensive parametric study is conducted to examine the effects of the external electric potential, the nonlocal parameter, the volume fraction of nano-voids, the temperature rise on the vibration of porous FGPM cylindrical nanoshells.

## 1. Introduction

Piezoelectric materials are characterized by the excellent coupling between the electric and mechanical fields. Applying mechanical load to piezoelectric materials generates an electric field, while putting piezoelectric materials in an electric field creates mechanical strain in them. This two-way property has made piezoelectric materials ideal for making actuators and sensors [1,2,3,4]. Besides, the two-way action of turning mechanical energy to electric energy and vice versa has made piezoelectric materials useful in resonant ultrasonic inspection and micro/nano piezoelectric power generators [5,6,7].

Unfortunately, there are some deficiencies such as low resistance to external loads, creeping in high temperature, and high stress concentration in homogeneous piezoelectric materials. In order to eliminate these problems, functionally graded piezoelectric materials (FGPMs) were proposed. The concept of functionally graded materials was first proposed in the 1980s [8]. Functionally graded materials are generally composed of two different materials, and are characterized by continuous variations in both mechanical properties and material composition in one or more dimension(s). Likewise, FGPMs are generally composed of two different piezoelectric materials. They have many advantages such as multifunctionality, ability to control deformation, and minimization or removal of stress. Hence, FGPMs have received wide engineering applications [9,10,11,12,13]. In FGPMs, owing to the technical issues, nano-voids or porosities may occur within materials. It is reported that a considerable number of nanopores appeared in the functionally graded material during the preparation process by the non-pressure sintering technique [14]. Thus, it is necessary to consider the porosity effect on vibration characteristics of porous FGPM structures.

With the rapid development in nanotechnology, the FGPMs have potential to be used in functional and structural elements in micro/nano electromechanical systems. It is known that FGPM nanostructures possess significant mechanical, thermal, electrical, and other physical properties.

Piezoelectric nanostructures have the dimension ranging from a few nanometers to several hundred nanometers. On this scale, the size effect was observed in both experiments and simulations [15,16,17,18]. One of effective nonclassical continuum theories considering size effect for piezoelectric nanostructures is Eringen’s nonlocal theory [19,20,21]. Ke et al. [22] used this theory to analyze free vibration of piezoelectric nanobeams subjected to thermo-mechanical-electro loading. Afterwards, the vibration of functionally graded piezoelectric nanoplates using the nonlocal elasticity theory was studied by Jandaghian and Rahmani [23]. The thermo-mechanical-electric vibration of FGPM nanoplates was studied by Jandaghian and Rahmani [24]. The vibration and buckling analyses of the piezoelectric nanobeams were carried out by Liang et al. [25]. Yan and Jiang [26] studied the surface effects on the vibration and buckling of the piezoelectric nanoplates. It is noted that all the above-mentioned studies concentrated on the piezoelectric nano beams or plates.

Cylindrical nanoshells possess specific functions in micro/nano electromechanical system. The size-dependent dynamic analysis of nanoshells, however, is limited in the open literature. Among them, the free vibration of magneto-electro-elastic cylindrical nanoshells was investigated by Ghadiri and Safarpour [27]. Fang et al. [28] conducted the free vibration analyses of piezoelectric nano double-shells. The instability and vibration of functionally graded nanoshells with internal fluid flow were analyzed by Ansari et al. [29]. In framework of the nonlocal elasticity theory, Sun et al. [30] analyzed the bucking of functionally graded cylindrical nanoshells. Ke et al. [31] studied the free vibration of piezoelectric nanoshells under an electric voltage.

In this article, vibration behavior of porous FGPM nanoshells subjected to the thermal and electrical loads is studied for the first time. Governing equations are derived from Hamilton’s principle by using the nonlocal elasticity theory and Love’s thin shell theory. Then, natural frequencies of the nanoshells are evaluated by the Navier technique and the Galerkin technique. Detailed results are shown to explore the influences of several key factors on vibration characteristics of FGPM nanoshells with nano-voids.

## 2. Preliminaries

### 2.1. Nonlocal Elasticity Theory for FGPMs

In Eringen’s nonlocal elastic theory [19,20,21], nonlocal constitutive equations are written as [19,32]:

(1) $$\sigma_{ij}=\int_{V}^{}\alpha_{0}\left(\left|x^{\prime }-x\right|,\frac{e_{0}a}{l_{e}}\right)\left[c_{ijkl}\varepsilon_{kl}(x^{\prime })-e_{kij}E_{k}(x^{\prime })-\beta_{ij}\Delta T\right]dx\prime$$

(2) $$D_{i}=\int_{V}^{}\alpha_{0}\left(\left|x^{\prime }-x\right|,\frac{e_{0}a}{l_{e}}\right)\left[e_{ikl}\varepsilon_{kl}(x^{\prime })+s_{ik}E_{k}(x^{\prime })+p_{i}\Delta T\right]dx^{\prime }$$

(3) $$\sigma_{ij,j}=\rho {\ddot{u}}_{i}\;D_{i,i}=0$$

(4) $$E_{i}=-{\tilde{\Phi }}_{,i}\;\varepsilon_{ij}=\frac{1}{2}(u_{i,j}+u_{j,i})$$

in which *i*, *j*, *l*, *k* = 1, 2, 3; *ε_ij_*, *σ_ij_*, *u_i_*, *E_i_* and *D_i_* denote the components of the strain, stress, displacement, electric field, and electric displacement, respectively; *e_kij_*, *c_ijkl_*, *p_i_*, *β_ij_* and *s_ik_* represent the components of the piezoelectric tensor, elasticity tensor, pyroelectric vector, thermal modulus tensor and the dielectric tensor, respectively; denotes the mass density; $\tilde{\Phi }$ and Δ*T* are the electric potential and temperature change, respectively; $\alpha_{0}\left(\left|x^{\prime }-x\right|,e_{0}a/l_{e}\right)$ is the nonlocal kernel function; $e_{0}a/l_{e}$ represents the scale parameter; *x’* represents all material point coordinates except *x* point in the area; $\left|x^{\prime }-x\right|$ represents the Euclidean Distance.

Equivalent differential forms can be used to represent the overall constitutive relation as follows [20]:

(5) $$\sigma_{ij}-{\left(e_{0}a\right)}^{2}\nabla^{2}\sigma_{ij}=c_{ijkl}\varepsilon_{kl}-e_{kij}E_{k}-\beta_{ij}\Delta T$$

(6) $$D_{i}-{\left(e_{0}a\right)}^{2}\nabla^{2}D_{i}=e_{ikl}\varepsilon_{kl}+s_{ik}E_{k}+p_{i}\Delta T$$

in which $\nabla^{2}$ is the Laplace Operator.

### 2.2. Nonlocal Porous FGPM Cylindrical Nanoshell Model

**Figure 1:**
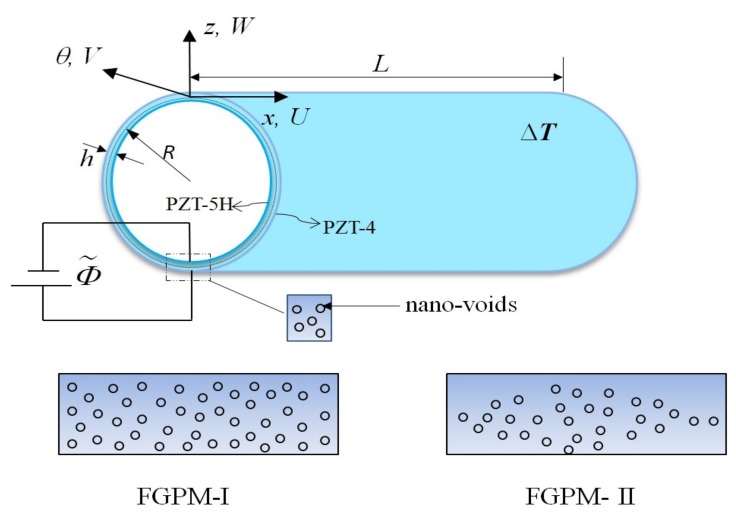
Schematic of a porous functionally graded piezoelectric material (FGPM) circular cylindrical nanoshell.

Consider a porous FGPM cylindrical nanoshell composed of PZT-5H and PZT-4. Figure 1 shows the geometry of the nanoshell with the thickness *h*, the middle-surface radius *R* and the length *L*. The FGPM nanoshell is supposed to contain nano-voids that disperse evenly (FGPM-I) or unevenly (FGPM-II) along the thickness direction. Additionally, the nanoshell is subjected to a uniform temperature change Δ*T* and electric potential $\tilde{\Phi }(x,\theta ,z,t)$. *U*(*x*, *θ t*), *V*(*x*, *θ*, *t*) and *W*(*x*, *θ*, *t*) are displacements of points at the middle plane of the shell in *x*-, *θ*- and *z*-axes directions, respectively.

The sum of PZT-5H and PZT-4 volume fractions is *V*_4_ + *V*_5H_ = 1 [33]; For PZT-4, the volume fraction can be written as [34,35,36]:

(7) $$V_{4}={\left(\frac{2z+h}{2h}\right)}^{N}$$

where the parameter *N* ∈ [0, ∞) represents the power-law index.

For the FGPM-I nanoshell, the general material properties are given by [37]

(8) $$P(z)=(P_{4}-P_{5H}){\left(\frac{z}{h}+\frac{1}{2}\right)}^{N}+P_{5H}-(P_{4}+P_{5H})\frac{\alpha }{2}$$

where *z* is the distance from the mid-surface of the FGPM cylindrical nanoshell; *P*_4_ and *P*_5H_ are material properties of PZT-4 and PZT-5H, respectively; *α* is the porosity volume fraction.

Therefore, the elastic constants *c_ij_*, the piezoelectric constants *e_ij_*, the mass density *ρ*, and the dielectric constants *s_ij_* of the FGPM-I nanoshell can be expressed as:

(9) $$\begin{array}{l} c_{ij}(z)=\left(c_{4ij}-c_{5Hij}\right){\left(\frac{z}{h}+\frac{1}{2}\right)}^{N}+c_{5Hij}-\left(c_{4ij}+c_{5Hij}\right)\frac{\alpha }{2}\\ \left(i,j)=\{(1,1),(1,2),(1,3),(2,2),(2,3),(3,3),(6,6)\right\} \end{array}$$

(10) $$\begin{array}{l} e_{ij}(z)=\left(e_{4ij}-e_{5Hij}\right){\left(\frac{z}{h}+\frac{1}{2}\right)}^{N}+e_{5Hij}-\left(e_{4ij}+e_{5Hij}\right)\frac{\alpha }{2}\\ \left(i,j)=\{(3,1),(3,2),(3,3)\right\} \end{array}$$

(11) $$\begin{array}{l} s_{ij}(z)=\left(s_{4ij}-s_{5Hij}\right){\left(\frac{z}{h}+\frac{1}{2}\right)}^{N}+s_{5Hij}-\left(s_{4ij}+s_{5Hij}\right)\frac{\alpha }{2}\\ \left(i,j)=\{(1,1),(3,3)\right\} \end{array}$$

(12) $$\rho (z)=\left(\rho_{4}-\rho_{5H}\right){\left(\frac{z}{h}+\frac{1}{2}\right)}^{N}+\rho_{5H}-\left(\rho_{4}+\rho_{5H}\right)\frac{\alpha }{2}$$

For the FGPM-II nanoshell, on the other hand, the material properties in Equations (9)–(12) can be replaced by [38]

(13) $$\begin{array}{l} c_{ij}(z)=\left(c_{4ij}-c_{5Hij}\right){\left(\frac{z}{h}+\frac{1}{2}\right)}^{N}+c_{5Hij}-\frac{\alpha }{2}\left(c_{4ij}+c_{5Hij}\right)\left(1-\frac{2\left|z\right|}{h}\right)\\ \left(i,j)=\{(1,1),(1,2),(1,3),(2,2),(2,3),(3,3),(6,6)\right\} \end{array}$$

(14) $$\begin{array}{l} e_{ij}(z)=\left(e_{4ij}-e_{5Hij}\right){\left(\frac{z}{h}+\frac{1}{2}\right)}^{N}+e_{5Hij}-\frac{\alpha }{2}\left(e_{4ij}+e_{5Hij}\right)\left(1-\frac{2\left|z\right|}{h}\right)\\ \left(i,j)=\{(3,1),(3,2),(3,3)\right\} \end{array}$$

(15) $$\begin{array}{l} s_{ij}(z)=\left(s_{4ij}-s_{5Hij}\right){\left(\frac{z}{h}+\frac{1}{2}\right)}^{N}+s_{5Hij}-\frac{\alpha }{2}\left(s_{4ij}+s_{5Hij}\right)\left(1-\frac{2\left|z\right|}{h}\right)\\ \left(i,j)=\{(1,1),(3,3)\right\} \end{array}$$

(16) $$\rho (z)=\left(\rho_{4}-\rho_{5H}\right){\left(\frac{z}{h}+\frac{1}{2}\right)}^{N}+\rho_{5H}-\frac{\alpha }{2}\left(\rho_{4}+\rho_{5H}\right)\left(1-\frac{2\left|z\right|}{h}\right)$$

According to the Kirchhoff–Love hypothesis, the displacement fields are [39]:

(17) $$u(x,\theta ,z,t)=U(x,\theta ,t)-z\frac{\partial W(x,\theta ,t)}{\partial x}$$

(18) $$v(x,\theta ,z,t)=V(x,\theta ,t)-\frac{z}{R}\frac{\partial W(x,\theta ,t)}{\partial \theta }$$

(19) w(x,θ,z,t)=W(x,θ,t)

in which *t* is time, and u(x,θ,z,t), v(x,θ,z,t) and w(x,θ,z,t) are the displacements of an arbitrary point along the *x*-, *θ*- and *z*-axes, respectively.

Using Love’s first approximation shell theory, the strain-displacement relations can be written as [40]:

(20) $$\varepsilon_{xx}=\frac{\partial U}{\partial x}-z\frac{\partial^{2}W}{\partial x^{2}}$$

(21) $$\varepsilon_{\theta \theta }=\frac{1}{R}\frac{\partial V}{\partial \theta }+\frac{W}{R}-\frac{z}{R^{2}}\left(\frac{\partial^{2}W}{\partial \theta^{2}}-\frac{\partial V}{\partial \theta }\right)$$

(22) $$\gamma_{x\theta }=\frac{\partial V}{\partial x}+\frac{1}{R}\frac{\partial U}{\partial \theta }-\frac{z}{R}\left(\frac{2\partial^{2}W}{\partial \theta \partial x}-\frac{\partial V}{\partial x}\right)$$

Following Wang [41], the distribution of electric potential along the thickness of the FGPM nanoshell is assumed as:

(23) $$\tilde{\Phi }(x,\theta ,z,t)=-\cos\left(\beta z\right)\Phi (x,\theta ,t)+\frac{2zV_{0}}{h}$$

in which β=π/h; *V*_0_ represents the initial external electric voltage applied to the FGPM nanoshell; *Φ*(*x*, *θ*, *t*) represents the spatial and time variation of the electric potential in the *x*-direction and *θ*-direction.

Using Equation (23), the electric field components *E_i_* are given by

(24) $$E_{x}=-{\tilde{\Phi }}_{,x}=\cos\left(\beta z\right)\frac{\partial \Phi }{\partial x}$$

(25) $$E_{\theta }=-\frac{1}{R+z}{\tilde{\Phi }}_{,\theta }=\frac{1}{R+z}\cos\left(\beta z\right)\frac{\partial \Phi }{\partial \theta }$$

(26) $$E_{z}=-{\tilde{\Phi }}_{,z}=-\beta \sin\left(\beta z\right)\Phi -\frac{2V_{0}}{h}$$

For the porous FGPM cylindrical nanoshell, the nonlocal constitutive relationship (5) and (6) can be given by [42,43]

(27) $$\left[1-{\left(e_{0}a\right)}^{2}\nabla^{2}\right]\left[\begin{array}{c} \sigma_{x}\\ \sigma_{\theta }\\ \sigma_{x\theta }\\ D_{x}\\ D_{\theta }\\ D_{z} \end{array}\right]=\left[\begin{array}{c} {\tilde{c}}_{11}\varepsilon_{xx}+{\tilde{c}}_{12}\varepsilon_{\theta \theta }-{\tilde{e}}_{31}E_{z}-{\tilde{\beta }}_{11}\Delta T\\ {\tilde{c}}_{12}\varepsilon_{xx}+{\tilde{c}}_{22}\varepsilon_{\theta \theta }-{\tilde{e}}_{32}E_{z}-{\tilde{\beta }}_{22}\Delta T\\ {\tilde{c}}_{66}\gamma_{x\theta }\\ {\tilde{s}}_{11}E_{x}\\ {\tilde{s}}_{22}E_{\theta }\\ {\tilde{e}}_{31}\varepsilon_{xx}+{\tilde{e}}_{32}\varepsilon_{\theta \theta }+{\tilde{s}}_{33}E_{z}+{\tilde{p}}_{3}\Delta T \end{array}\right]$$

in which $\nabla^{2}=\partial^{2}/\partial x^{2}+\partial^{2}/\partial {\left(R\theta \right)}^{2}$; ${\tilde{c}}_{ij}$, ${\tilde{e}}_{ij}$, ${\tilde{s}}_{ij}$, ${\tilde{\beta }}_{11}$, ${\tilde{\beta }}_{22}$ and ${\tilde{p}}_{3}$ are defined as:

(28) $$\begin{array}{l} {\tilde{c}}_{11}=c_{11}-\frac{c_{13}{}^{2}}{c_{33}},\;{\tilde{c}}_{12}=c_{12}-\frac{c_{13}{}^{2}}{c_{33}},\;{\tilde{c}}_{22}=c_{22}-\frac{c_{23}{}^{2}}{c_{33}},\;{\tilde{c}}_{66}=c_{66},\;\\ \;{\tilde{s}}_{11}=s_{11},\;{\tilde{s}}_{22}={\tilde{s}}_{11},\;{\tilde{s}}_{33}=s_{33}+\frac{e_{33}{}^{2}}{c_{33}},\;{\tilde{\beta }}_{11}=\beta_{11}-\frac{c_{13}\beta_{33}}{c_{33}},\;{\tilde{\beta }}_{22}={\tilde{\beta }}_{11}\\ \;{\tilde{p}}_{3}=p_{3}+\frac{e_{33}\beta_{33}}{c_{33}},\;{\tilde{e}}_{31}=e_{31}-\frac{c_{13}e_{33}}{c_{33}},\;{\tilde{e}}_{32}=e_{32}-\frac{c_{23}e_{33}}{c_{33}} \end{array}$$

The strain energy $\Pi_{s}$ of the porous FGPM cylindrical nanoshell is expressed as follows:

(29) $$\Pi_{s}=\frac{1}{2}\int_{0}^{L}\int_{0}^{2\pi }\int_{-\frac{h}{2}}^{\frac{h}{2}}(\sigma_{x}\varepsilon_{xx}+\sigma_{\theta }\varepsilon_{\theta \theta }+\sigma_{x\theta }\gamma_{x\theta }-D_{x}E_{x}-D_{\theta }E_{\theta }-D_{z}E_{z})Rdzd\theta dx$$

Substituting Equations (20)–(22) and Equations (24)–(26) into Equation (29) gives

(30) $$\begin{array}{l} \Pi_{s}=\frac{1}{2}\int_{0}^{L}\int_{0}^{2\pi }\int_{-\frac{h}{2}}^{\frac{h}{2}}[N_{x}\frac{\partial U}{\partial x}+\frac{N_{\theta }}{R}\left(\frac{\partial V}{\partial x}+W\right)+N_{x\theta }\left(\frac{\partial V}{\partial x}+\frac{1}{R}\frac{\partial U}{\partial \theta }\right)-M_{x}\frac{\partial^{2}W}{\partial x^{2}}\\ -\frac{M_{\theta }}{R^{2}}\left(\frac{\partial^{2}W}{\partial \theta^{2}}-\frac{\partial V}{\partial \theta }\right)-\frac{M_{x\theta }}{R}\left(\frac{2\partial^{2}W}{\partial x\partial \theta }-\frac{\partial V}{\partial x}\right)]Rdzd\theta dx\\ -\frac{1}{2}\int_{0}^{L}\int_{0}^{2\pi }\int_{-\frac{h}{2}}^{\frac{h}{2}}\left[D_{x}\cos(\beta z)\frac{\partial \Phi }{\partial x}+D_{\theta }\frac{\cos(\beta z)}{R+z}\frac{\partial \Phi }{\partial \theta }-D_{z}\left(\beta \sin(\beta z)\Phi +\frac{2V_{0}}{h}\right)\right]Rdzd\theta dx \end{array}$$

in which the resultant forces and the moments can be respectively calculated as

(31) $$\left\{N_{x},N_{\theta },N_{x\theta }\right\}=\int_{-\frac{h}{2}}^{\frac{h}{2}}\left\{\sigma_{x},\sigma_{\theta },\sigma_{x\theta }\right\}dz$$

(32) $$\left\{M_{x},M_{\theta },M_{x\theta }\right\}=\int_{-\frac{h}{2}}^{\frac{h}{2}}\left\{\sigma_{x},\sigma_{\theta },\sigma_{x\theta }\right\}zdz$$

The kinetic energy $\Pi_{k}$ is given by:

(33) $$\Pi_{k}=\frac{1}{2}\int_{0}^{L}\int_{0}^{2\pi }I_{1}\left[{\left(\frac{\partial U}{\partial t}\right)}^{2}+{\left(\frac{\partial V}{\partial t}\right)}^{2}+{\left(\frac{\partial W}{\partial t}\right)}^{2}\right]Rd\theta dx$$

in which $I_{1}=\int_{-h/2}^{h/2}\rho (z)dz$, and the rotatory inertia term is neglected due to its slight impact.

Moreover, the work $\Pi_{F}$ done by external forces can be written as:

(34) $$\Pi_{F}=\frac{1}{2}\int_{0}^{L}\int_{0}^{2\pi }\left[\left(N_{Tx}+N_{Ex}\right){\left(\frac{\partial W}{\partial x}\right)}^{2}+\frac{N_{T\theta }+N_{E\theta }}{R^{2}}{\left(\frac{\partial W}{\partial \theta }\right)}^{2}\right]Rd\theta dx$$

in which ($N_{Ex}$,$N_{E\theta }$)and ($N_{Tx}$,$N_{T\theta }$) are the electrical and thermal forces induced by the uniform external electric voltage *V*_0_ and uniform temperature rise Δ*T*, respectively. They are given by

(35) $$\left\{N_{Tx},N_{T\theta }\right\}=\int_{-\frac{h}{2}}^{\frac{h}{2}}\left\{{\tilde{\beta }}_{11},{\tilde{\beta }}_{22}\right\}\Delta Tdz,\;\left\{N_{Ex},N_{E\theta }\right\}=\frac{1}{h}\int_{-\frac{h}{2}}^{\frac{h}{2}}-2\left\{{\tilde{e}}_{31},{\tilde{e}}_{32}\right\}V_{0}dz$$

Using Hamilton’s principle [44,45]:

(36) $$\int_{0}^{t}\left[\delta \Pi_{k}-\delta \Pi_{s}-\delta \Pi_{F}\right]dt=0$$

and applying Equations (29), (33), and (34), it yields the governing equations:

(37) $$\frac{\partial N_{x}}{\partial x}+\frac{1}{R}\frac{\partial N_{x\theta }}{\partial \theta }-I_{1}\frac{\partial^{2}U}{\partial t^{2}}=0$$

(38) $$\frac{\partial N_{x\theta }}{\partial x}+\frac{1}{R}\frac{\partial N_{\theta }}{\partial \theta }+\frac{1}{R}\frac{\partial M_{x\theta }}{\partial x}+\frac{1}{R^{2}}\frac{\partial M_{\theta }}{\partial \theta }-I_{1}\frac{\partial^{2}V}{\partial t^{2}}=0$$

(39) $$\frac{\partial^{2}M_{x}}{\partial x^{2}}+\frac{2}{R}\frac{\partial^{2}M_{x\theta }}{\partial x\partial \theta }+\frac{1}{R^{2}}\frac{\partial^{2}M_{\theta }}{\partial \theta^{2}}-\frac{N_{\theta }}{R}-N_{x1}\frac{\partial^{2}W}{\partial x^{2}}-\frac{N_{\theta 1}}{R^{2}}\frac{\partial^{2}W}{\partial \theta^{2}}-I_{1}\frac{\partial^{2}W}{\partial t^{2}}=0$$

(40) $$\int_{-\frac{h}{2}}^{\frac{h}{2}}\left[\frac{\partial D_{x}}{\partial x}\cos\left(\beta z\right)+\frac{\cos\left(\beta z\right)}{R+z}\frac{\partial D_{\theta }}{\partial \theta }+D_{z}\beta \sin\beta z\right]dz=0$$

where

(41) $$N_{x1}=N_{Tx}+N_{Ex},\;N_{\theta 1}=N_{T\theta }+N_{E\theta }$$

The corresponding boundary conditions are:

(42) $$\begin{array}{ccc} U=0 & \mathrm{or} & N_{x} \end{array}n_{x}+\frac{N_{x\theta }}{R}n_{\theta }=0$$

(43) $$\begin{array}{ccc} V=0 & \mathrm{or} & \left(N_{x\theta }+\frac{M_{x\theta }}{R}\right)n_{x}+\left(\frac{N_{\theta }}{R}+\frac{M_{\theta }}{R^{2}}\right)n_{\theta }=0 \end{array}$$

(44) $$\begin{array}{l} \begin{array}{ccc} W=0 & \mathrm{or} & n_{x} \end{array}\left(\frac{\partial M_{x}}{\partial x}+\frac{1}{R}\frac{\partial M_{x\theta }}{\partial \theta }-N_{x1}\frac{\partial W}{\partial x}\right)\\ +n_{\theta }\left(\frac{1}{R^{2}}\frac{\partial M_{\theta }}{\partial \theta }+\frac{1}{R}\frac{\partial M_{x\theta }}{\partial x}-\frac{N_{\theta 1}}{R^{2}}\frac{\partial W}{\partial \theta }\right)=0 \end{array}$$

(45) $$\begin{array}{ccc} \frac{\partial W}{\partial x}=0 & \mathrm{or} & M_{x} \end{array}n_{x}+\frac{M_{x\theta }}{R}n_{\theta }=0$$

(46) $$\begin{array}{ccc} \frac{\partial W}{\partial \theta }=0 & \mathrm{or} & \frac{M_{x\theta }}{R} \end{array}n_{x}+\frac{M_{\theta }}{R^{2}}n_{\theta }=0$$

(47) $$\begin{array}{ccc} \Phi =0 & \mathrm{or} & \int_{-\frac{h}{2}}^{\frac{h}{2}}\left[\cos(\beta z)D_{x}n_{x}+\frac{\cos(\beta z)}{R+z}D_{\theta }n_{\theta }\right]dz \end{array}=0$$

where *n_x_* and *n_θ_* denote the direction cosines of the outward unit normal to the boundaries of the mid-plane.

From Equation (27), we obtain the following equations:

(48) $$N_{x}-{\left(e_{0}a\right)}^{2}\nabla^{2}N_{x}=A_{11}\frac{\partial U}{\partial x}+\frac{A_{12}}{R}\left(\frac{\partial V}{\partial \theta }+W\right)-B_{11}\frac{\partial^{2}W}{\partial x^{2}}-\frac{B_{12}}{R^{2}}\frac{\partial^{2}W}{\partial \theta^{2}}+F_{31}\Phi -N_{x1}$$

(49) $$N_{\theta }-{\left(e_{0}a\right)}^{2}\nabla^{2}N_{\theta }=A_{12}\frac{\partial U}{\partial x}+\frac{A_{22}}{R}\left(\frac{\partial V}{\partial \theta }+W\right)-B_{12}\frac{\partial^{2}W}{\partial x^{2}}-\frac{B_{11}}{R^{2}}\frac{\partial^{2}W}{\partial \theta^{2}}+F_{32}\Phi -N_{\theta 1}$$

(50) $$N_{x\theta }-{\left(e_{0}a\right)}^{2}\nabla^{2}N_{x\theta }=A_{66}\left(\frac{\partial V}{\partial \theta }+\frac{1}{R}\frac{\partial U}{\partial \theta }\right)-\frac{2B_{66}}{R}\frac{\partial^{2}W}{\partial x\partial \theta }$$

(51) $$M_{x}-{\left(e_{0}a\right)}^{2}\nabla^{2}M_{x}=-D_{11}\frac{\partial^{2}W}{\partial x^{2}}-\frac{D_{12}}{R^{2}}\left(\frac{\partial^{2}W}{\partial \theta^{2}}-\frac{\partial V}{\partial \theta }\right)+B_{11}\frac{\partial U}{\partial x}+\frac{B_{12}}{R}\frac{\partial V}{\partial \theta }+E_{31}\Phi$$

(52) $$M_{\theta }-{\left(e_{0}a\right)}^{2}\nabla^{2}M_{\theta }=-D_{12}\frac{\partial^{2}W}{\partial x^{2}}-\frac{D_{22}}{R^{2}}\left(\frac{\partial^{2}W}{\partial \theta^{2}}-\frac{\partial V}{\partial \theta }\right)+B_{12}\frac{\partial U}{\partial x}+\frac{B_{11}}{R}\frac{\partial V}{\partial \theta }+E_{32}\Phi$$

(53) $$M_{x\theta }-{\left(e_{0}a\right)}^{2}\nabla^{2}M_{x\theta }=-\frac{D_{66}}{R}\left(\frac{2\partial^{2}W}{\partial x\partial \theta }-\frac{\partial V}{\partial x}\right)+B_{66}\left(\frac{1}{R}\frac{\partial U}{\partial \theta }+\frac{\partial V}{\partial x}\right)$$

(54) $$\int_{-\frac{h}{2}}^{\frac{h}{2}}\left[1-{\left(e_{0}a\right)}^{2}\nabla^{2}\right]D_{x}\cos\left(\beta z\right)dz=X_{11}\frac{\partial \Phi }{\partial x}$$

(55) $$\int_{-\frac{h}{2}}^{\frac{h}{2}}\left[1-{\left(e_{0}a\right)}^{2}\nabla^{2}\right]D_{\theta }\frac{\cos\left(\beta z\right)}{R+z}dz=\frac{X_{22}}{R}\frac{\partial \Phi }{\partial \theta }$$

(56) $$\begin{array}{l} \int_{-\frac{h}{2}}^{\frac{h}{2}}\left[1-{\left(e_{0}a\right)}^{2}\nabla^{2}\right]D_{z}\beta \sin(\beta z)dz=-E_{31}\frac{\partial^{2}W}{\partial x^{2}}-\frac{E_{32}}{R^{2}}\left(\frac{\partial^{2}W}{\partial \theta^{2}}-\frac{\partial V}{\partial \theta }\right)\\ \;+F_{31}\left(\frac{1}{R}\frac{\partial V}{\partial \theta }+\frac{\partial U}{\partial x}\right)-X_{33}\Phi \end{array}$$

where

$$\begin{array}{l} A_{11}=\int_{-\frac{h}{2}}^{\frac{h}{2}}{\tilde{c}}_{11}(z)dz,\;B_{11}=\int_{-\frac{h}{2}}^{\frac{h}{2}}{\tilde{c}}_{11}(z)zdz,\;D_{11}=\int_{-\frac{h}{2}}^{\frac{h}{2}}{\tilde{c}}_{11}(z)z^{2}dz\\ A_{12}=\int_{-\frac{h}{2}}^{\frac{h}{2}}{\tilde{c}}_{12}(z)dz,\;B_{12}=\int_{-\frac{h}{2}}^{\frac{h}{2}}{\tilde{c}}_{12}(z)zdz,\;D_{12}=\int_{-\frac{h}{2}}^{\frac{h}{2}}{\tilde{c}}_{12}(z)z^{2}dz\\ A_{22}=\int_{-\frac{h}{2}}^{\frac{h}{2}}{\tilde{c}}_{22}(z)dz,\;B_{22}=\int_{-\frac{h}{2}}^{\frac{h}{2}}{\tilde{c}}_{22}(z)zdz,\;D_{22}=\int_{-\frac{h}{2}}^{\frac{h}{2}}{\tilde{c}}_{22}(z)z^{2}dz\\ A_{66}=\int_{-\frac{h}{2}}^{\frac{h}{2}}{\tilde{c}}_{66}(z)dz,\;B_{66}=\int_{-\frac{h}{2}}^{\frac{h}{2}}{\tilde{c}}_{66}(z)zdz,\;D_{66}=\int_{-\frac{h}{2}}^{\frac{h}{2}}{\tilde{c}}_{66}(z)z^{2}dz\\ F_{31}=\int_{-\frac{h}{2}}^{\frac{h}{2}}{\tilde{e}}_{31}(z)\beta \sin(\beta z)dz\;E_{31}=\int_{-\frac{h}{2}}^{\frac{h}{2}}{\tilde{e}}_{31}(z)\beta z\sin(\beta z)dz\\ F_{32}=\int_{-\frac{h}{2}}^{\frac{h}{2}}{\tilde{e}}_{32}(z)\beta \sin(\beta z)dz\;E_{32}=\int_{-\frac{h}{2}}^{\frac{h}{2}}{\tilde{e}}_{32}(z)\beta z\sin(\beta z)dz\\ X_{11}=\int_{-\frac{h}{2}}^{\frac{h}{2}}{\tilde{s}}_{11}(z)\cos^{2}(\beta z)dz,\;X_{22}=\int_{-\frac{h}{2}}^{\frac{h}{2}}{\tilde{s}}_{22}(z){\left[\frac{\cos(\beta z)}{R+z}\right]}^{2}dz\\ X_{33}=\int_{-\frac{h}{2}}^{\frac{h}{2}}{\tilde{s}}_{33}(z){\left[\beta \sin(\beta z)\right]}^{2}dz \end{array}$$

Substituting Equations (48)–(56) into Equations (37)–(40) gives

(57) $$\begin{array}{l} A_{11}\frac{\partial^{2}U}{\partial x^{2}}+\frac{A_{12}}{R}\left(\frac{\partial^{2}V}{\partial x\partial \theta }+\frac{\partial W}{\partial x}\right)-B_{11}\frac{\partial^{3}W}{\partial x^{3}}-\frac{B_{12}}{R^{2}}\frac{\partial^{3}W}{\partial x\partial \theta^{2}}+F_{31}\frac{\partial \Phi }{\partial x}\\ \;+\frac{A_{66}}{R}\left(\frac{1}{R}\frac{\partial^{2}U}{\partial \theta^{2}}+\frac{\partial^{2}V}{\partial x\partial \theta }\right)-\frac{2B_{66}}{R^{2}}\frac{\partial^{3}W}{\partial x\partial \theta^{2}}-\left[1-{\left(e_{0}a\right)}^{2}\nabla^{2}\right]I_{1}\frac{\partial^{2}U}{\partial t^{2}}=0 \end{array}$$

(58) $$\begin{array}{l} A_{66}\left(\frac{1}{R}\frac{\partial^{2}U}{\partial x\partial \theta }+\frac{\partial^{2}V}{\partial x^{2}}\right)-\frac{2B_{66}}{R}\frac{\partial^{3}W}{\partial x^{2}\partial \theta }+\frac{A_{12}}{R}\frac{\partial^{2}U}{\partial x\partial \theta }+\frac{A_{22}}{R^{2}}\left(\frac{\partial^{2}V}{\partial \theta^{2}}+\frac{\partial W}{\partial \theta }\right)\\ -\frac{B_{12}}{R}\frac{\partial^{3}W}{\partial x^{2}\partial \theta }-\frac{B_{11}}{R^{3}}\frac{\partial^{3}W}{\partial \theta^{3}}+\frac{1}{R}F_{31}\frac{\partial \Phi }{\partial \theta }-\frac{D_{66}}{R^{2}}\left(2\frac{\partial^{3}W}{\partial x^{2}\partial \theta }-\frac{\partial^{2}V}{\partial x^{2}}\right)\\ +\frac{B_{66}}{R}\left(\frac{1}{R}\frac{\partial^{2}U}{\partial x\partial \theta }+\frac{\partial^{2}V}{\partial x^{2}}\right)-\frac{D_{12}}{R^{2}}\frac{\partial^{3}W}{\partial x^{2}\partial \theta }-\frac{D_{22}}{R^{4}}\left(\frac{\partial^{3}W}{\partial \theta^{3}}-\frac{\partial^{2}V}{\partial \theta^{2}}\right)\\ +\frac{E_{32}}{R^{2}}\frac{\partial \Phi }{\partial \theta }+\frac{B_{12}}{R^{2}}\frac{\partial^{2}U}{\partial x\partial \theta }+\frac{B_{11}}{R^{3}}\frac{\partial^{2}V}{\partial \theta^{2}}-\left[1-{\left(e_{0}a\right)}^{2}\nabla^{2}\right]I_{1}\frac{\partial^{2}V}{\partial t^{2}}=0 \end{array}$$

(59) $$\begin{array}{l} -D_{11}\frac{\partial^{4}W}{\partial x^{4}}-\frac{D_{12}}{R^{2}}\left(\frac{\partial^{4}W}{\partial x^{2}\partial \theta^{2}}-\frac{\partial^{3}V}{\partial x^{2}\partial \theta }\right)+E_{31}\frac{\partial^{2}\Phi }{\partial x^{2}}+B_{11}\frac{\partial^{3}U}{\partial x^{3}}+\frac{B_{12}}{R}\frac{\partial^{3}V}{\partial x^{2}\partial \theta }\\ +\frac{2}{R}\left[-\frac{D_{66}}{R}\left(2\frac{\partial^{4}W}{\partial x^{2}\partial \theta^{2}}-\frac{\partial^{3}V}{\partial x^{2}\partial \theta }\right)+B_{66}\left(\frac{1}{R}\frac{\partial^{3}U}{\partial x\partial \theta^{2}}+\frac{\partial^{3}V}{\partial x^{2}\partial \theta }\right)\right]\\ +\frac{1}{R^{2}}\left[-D_{12}\frac{\partial^{4}W}{\partial x^{2}\partial \theta^{2}}-\frac{D_{22}}{R^{2}}\left(\frac{\partial^{4}W}{\partial \theta^{4}}-\frac{\partial^{3}V}{\partial \theta^{3}}\right)+E_{32}\frac{\partial^{2}\Phi }{\partial \theta^{2}}+B_{12}\frac{\partial^{3}U}{\partial x\partial \theta^{2}}+\frac{B_{11}}{R}\frac{\partial^{3}V}{\partial \theta^{3}}\right]\\ -\frac{1}{R}\left[A_{12}\frac{\partial U}{\partial x}+\frac{A_{22}}{R}\left(\frac{\partial V}{\partial \theta }+W\right)-B_{12}\frac{\partial^{2}W}{\partial x^{2}}-\frac{B_{11}}{R^{2}}\frac{\partial^{2}W}{\partial \theta^{2}}+F_{31}\Phi \right]\\ -\left[1-{\left(e_{0}a\right)}^{2}\nabla^{2}\right]\left(N_{x1}\frac{\partial^{2}W}{\partial x^{2}}+\frac{N_{\theta 1}}{R^{2}}\frac{\partial^{2}W}{\partial \theta^{2}}\right)-\left[1-{\left(e_{0}a\right)}^{2}\nabla^{2}\right]I_{1}\frac{\partial^{2}W}{\partial t^{2}}=0 \end{array}$$

(60) $$X_{11}\frac{\partial^{2}\Phi }{\partial x^{2}}+\frac{X_{22}}{R^{2}}\frac{\partial^{2}\Phi }{\partial \theta^{2}}-X_{33}\Phi -\frac{E_{32}}{R^{2}}\left(\frac{\partial^{2}W}{\partial \theta^{2}}-\frac{\partial V}{\partial \theta }\right)+F_{31}\left(\frac{\partial U}{\partial x}+\frac{1}{R}\frac{\partial V}{\partial \theta }\right)-E_{31}\frac{\partial^{2}W}{\partial x^{2}}=0$$

The electric potential at both ends of the FGPM nanoshell is assumed to be zero. Then, the associated boundary conditions are expressed as

(61) $$V=W=\Phi =\frac{\partial U}{\partial x}=\frac{\partial^{2}W}{\partial x^{2}}=\;0$$

for a simply-supported end, and

(62) $$U=V=W=\Phi =\frac{\partial W}{\partial x}=0$$

for a clamped end.

## 3. Solution Procedure

### 3.1. Navier Procedure

For the porous FGPM cylindrical nanoshell with simply supported-simply supported (SS-SS) boundary condition, analytical solutions of the free vibration problem can be obtained utilizing Navier’s method. For this purpose, the following displacement functions which satisfy the SS-SS boundary condition are introduced:

(63) $$U(x,\theta ,t)=U_{mn}\cos\left(\frac{m\pi x}{L}\right)\cos(n\theta )e^{i\omega t}$$

(64) $$V(x,\theta ,t)=V_{mn}\sin\left(\frac{m\pi x}{L}\right)\sin(n\theta )e^{i\omega t}$$

(65) $$W(x,\theta ,t)=W_{mn}\sin\left(\frac{m\pi x}{L}\right)\cos(n\theta )e^{i\omega t}$$

(66) $$\Phi (x,\theta ,t)=\Phi_{mn}\sin\left(\frac{m\pi x}{L}\right)\cos(n\theta )e^{i\omega t}$$

where $U_{mn}$, $V_{mn}$, $W_{mn}$ and $\Phi_{mn}$ represent the displacement amplitude components; *m* and *n* are mode numbers; *ω* represents the natural circular frequency of the porous FGPM nanoshell.

Substituting Equations (63)–(66) into Equations (57)–(60), the following equation can be obtained

(67) $$\left[\begin{array}{cccc} q_{11} & q_{12} & q_{13} & q_{14}\\ q_{21} & q_{22} & q_{23} & q_{24}\\ q_{31} & q_{32} & q_{33} & q_{34}\\ q_{41} & q_{42} & q_{43} & q_{44} \end{array}\right]\left\{\begin{array}{c} U_{mn}\\ V_{mn}\\ W_{mn}\\ \Phi_{mn} \end{array}\right\}=\left\{\begin{array}{c} 0\\ 0\\ 0\\ 0 \end{array}\right\}$$

The elements in the above matrix are given in the Appendix A Equation (A1). Equation (67) gives the characteristic equation for the natural frequencies of the porous FGPM cylindrical nanoshell. To obtain a nontrivial solution, the determinant of the coefficient matrix must be set to zero.

### 3.2. Galerkin Solution

For clamped-simply supported (C-SS) and clamped-clamped (C-C) boundary conditions, the spatial displacement field of the porous FGPM nanoshell is expressed as [46]:

(68) $$U(x,\theta ,t)=U_{mn}\frac{\partial \phi \left(x\right)}{\partial x}\cos(n\theta )e^{i\omega t}$$

(69) $$V(x,\theta ,t)=V_{mn}\;\phi \left(x\right)\sin(n\theta )e^{i\omega t}$$

(70) $$W(x,\theta ,t)=W_{mn}\;\phi \left(x\right)\cos(n\theta )e^{i\omega t}$$

(71) $$\Phi (x,\theta ,t)=\Phi_{mn}\;\phi \left(x\right)\cos(n\theta )e^{i\omega t}$$

Thereinto, the axial modal beam function *ϕ*(*x*) could be written as:

(72) $$\phi \left(x\right)=c_{1}\cosh\left(\frac{\lambda_{i}x}{L}\right)+c_{2}\cos\left(\frac{\lambda_{i}x}{L}\right)-\zeta_{i}\left[c_{3}\sinh\left(\frac{\lambda_{i}x}{L}\right)+c_{4}\sin\left(\frac{\lambda_{i}x}{L}\right)\right]$$

where the constants *c*_1_, *c*_2_, *c*_3_, *c*_4_, *ζ_i_* and *λ_i_* (*i* = 1, 2, 3, 4…) are given in Table 1.

Inserting Equations (68)–(71) in Equations (57)–(60) and applying the Galerkin method, we obtain:

(73) $$\left(K-\omega^{2}M\right)\left\{\begin{array}{c} U_{mn}\\ V_{mn}\\ W_{mn}\\ \Phi_{mn} \end{array}\right\}=\left\{\begin{array}{c} 0\\ 0\\ 0\\ 0 \end{array}\right\}$$

in which the matrices **M** and **K** are the mass matrix and stiffness matrix of the porous FGPM cylindrical nanoshell, respectively.

To find the non-zero solutions, the determinant of the coefficient matrix must be equal to zero. Then, natural frequencies of FGPM nanoshells with nano-voids can be determined [47,48,49,50,51,52,53,54].

## 4. Results and Discussion

For examining the validity of the present analysis, the comparison is performed on natural frequencies of a PZT-4 piezoelectric cylindrical nanoshell. Table 2, Table 3 and Table 4 list the natural frequencies of the piezoelectric nanoshell under different boundary conditions with *h* = 1 nm, *R*/*h* = 50, *L*/*R* = 12, *m* = 1, Δ*T* = 0, and *V*_0_ = 0. Material properties of PZT-4 are shown in Table 5. It is found that the present results match those given by Ke et al. [31] very well, bespeaking the validity of the present study.

In the following sections, free vibration of the porous FGPM cylindrical nanoscale shell shown in Figure 1 is performed; the material properties of the nanoshell are displayed in Table 5. If not specified, the following parameters are used:

*h* = 0.1 nm, *R/h* = 50, *L/R* = 6, *m* = 1, *N* = 1, *α* = 0.1, *V*_0_ = 0, Δ*T* = 0, *e*_0_*a* = 2 nm

In Table 6, Table 7 and Table 8, the variation of natural frequency of the FGPM-I nanoshell against the circumferential wave number is represented for different porosity volume fractions and different boundary conditions, where *N* = 20. Among them, α = 0 corresponds to the FGPM cylindrical nanoshell without nano-voids. The results reveal that the natural frequency decreases as the porosity volume fraction increases. With the increase of the circumferential wave number, it is seen that the natural frequency decreases first and then increases. In addition, under the same condition, the SS-SS porous FGPM nanoshell has the lowest natural frequency while the C-C one has the highest natural frequency. This is because the end support is the weakest (in terms of stiffness) for the SS-SS FGPM nanoshell and the strongest for the C-C one. Under the SS-SS boundary condition, it is seen that the minimum natural frequency occurs at *n* = 3. Therefore, the fundamental frequency of the SS-SS FGPM nanoshell corresponds to mode (*m* = 1, *n* = 3). In the next studies, the SS-SS FGPM nanoshell is taken as an example and the mode (1,3) is chosen as a representative mode.

Natural frequency against the radius-to-thickness ratio for different porosity volume fractions is plotted for the FGPM-I nanoshell in Table 9. As the radius-to-thickness ratio increases, one can see that the natural frequency decreases initially and then increases; the frequency does not change monotonously with the radius-to-thickness ratio.

**Figure 2:**
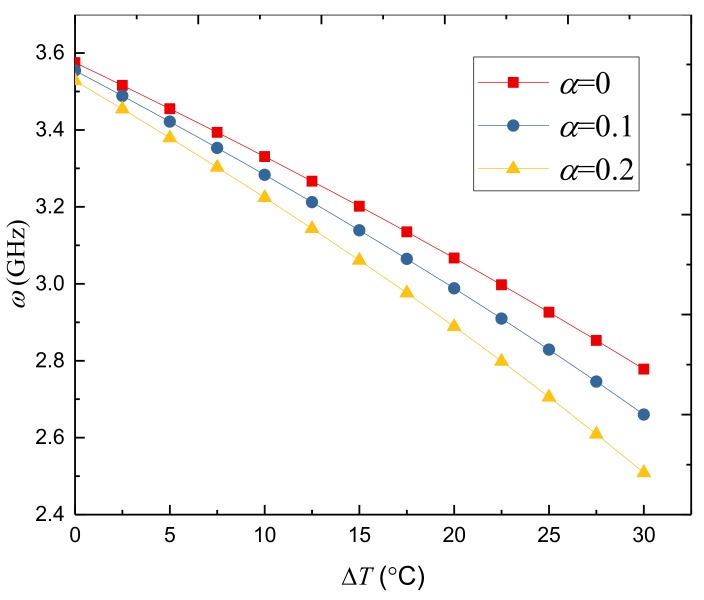
Variation of natural frequency *ω*(GHz) against temperature change △*T* (°C) for different porosity volume fractions of the FGPM-I nanoshell (*n* = 3, *N* = 20).

Figure 2 presents the effect of temperature change on the natural frequency of the FGPM-I nanoshell. The natural frequency decreases with the increase of temperature change. This is due to the fact that the larger temperature change results in a reduction in the stiffness and hence leads to the lower natural frequency of the porous FGPM nanoshell.

Table 10 illustrates the natural frequency against the circumferential wave number for different power-law indexes of the FGPM-I nanoshell. The natural frequencies of the FGPM nanoshell decreases with the increase of the power-law index. Additionally, it is seen that the fundamental natural frequency occurs at mode (*m* = 1, *n* = 3), which does not change with the power-law index.

**Figure 3:**
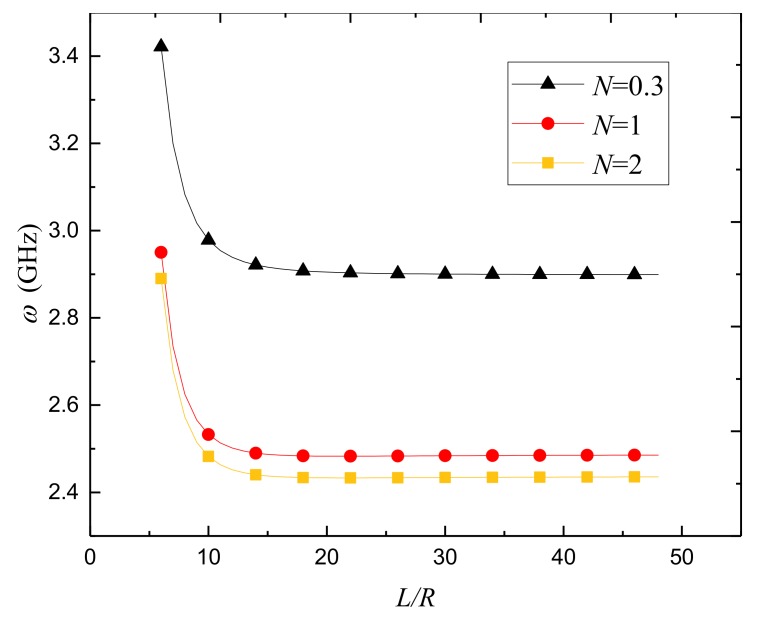
Variation of natural frequency *ω*(GHz) against length-to-radius ratio for different power-law indexes *N* of FGPM-I nanoshell (*n* = 3).

Figure 3 gives the variation of the natural frequency against the length-to-radius ratio for different power-law indexes. As a whole, it is observed that the natural frequency is quite susceptive to the length-to-radius ratio when this ratio is small; the frequency drops quickly as the length-to-radius ratio increases of the porous FGPM nanoshell. However, when *L*/*R* > 15, the natural frequency is no longer sensitive to the length-to-radius ratio change.

**Figure 4:**
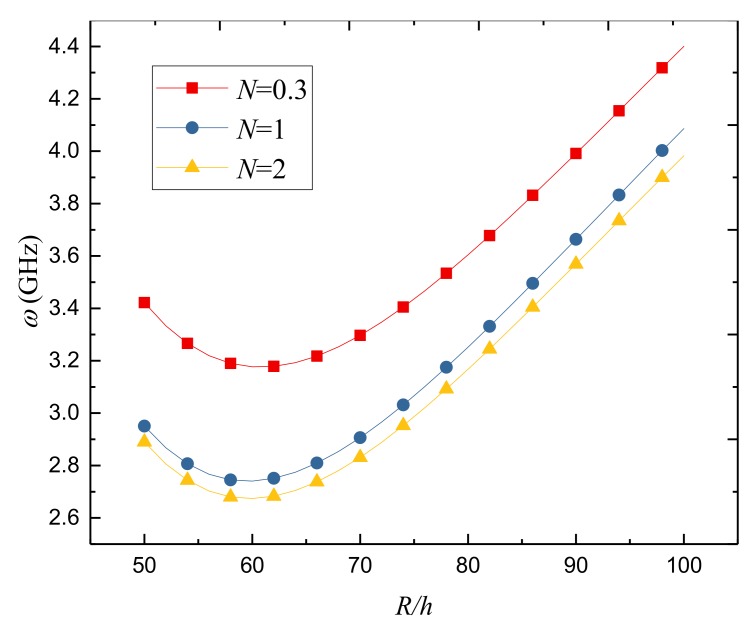
Variation of the natural frequency *ω*(GHz) against the radius-to-thickness ratio for different power-law indexes *N* of FGPM-I nanoshell (*n* = 3, *L* = 300 *h*).

Figure 4 presents the variation of natural frequency against the radius-to-thickness ratio for different power-law indexes. The natural frequency decreases first and then increases as the radius-to-thickness ratio increases. A trend can also be observed that the natural frequency decreases gradually with the increase of the power-law index.

**Figure 5:**
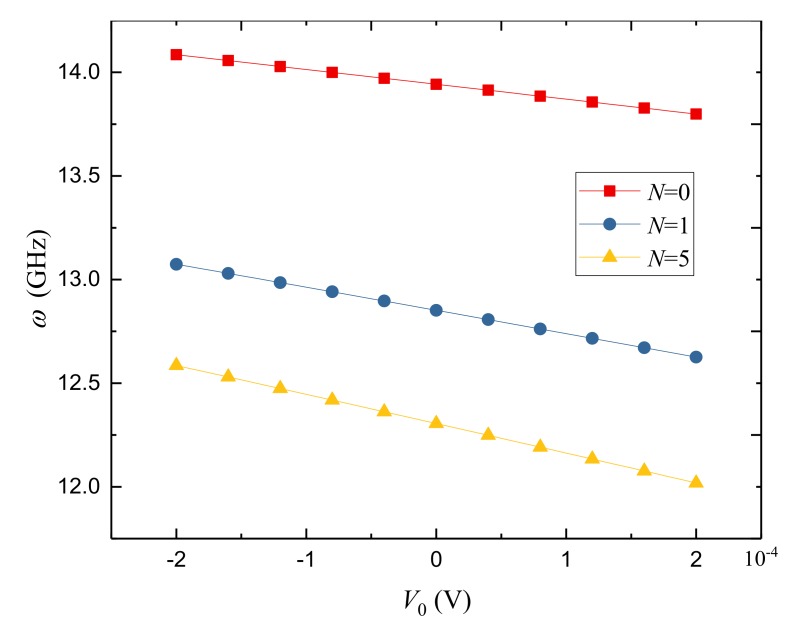
Variation of natural frequency *ω*(GHz) against external electric potential *V*_0_ for different power-law indexes of FGPM-I nanoshell (*n* = 1).

The variation of the natural frequency against external electric potential *V*_0_ for different power-law indexes is presented in Figure 5. Here, *N* = 0 corresponds to the cylindrical nanoshell made of pure PZT-4. As we can see, the natural frequency is quite sensitive to the applied external electric voltage. The natural frequency decreases as the voltage changes from *V*_0_ = −0.0002 V to 0.0002 V. The reason is that the axial and circumferential compressive and tensile forces are generated in the porous FGPM nanoshells by the applied positive and negative voltages, respectively. Thereinto, the applied positive voltage reduces the nanoshell stiffness but the negative voltage increases the nanoshell stiffness.

Table 11 presents the variation of nonlocal parameter against natural frequency of the FGPM-I nanoshell. One can see that the frequency decreases gradually with the increasing nonlocal parameter. This is because the nonlocal effect tends to decrease the stiffness of the nanoshell and hence decreases the natural frequency. This phenomenon was also found in nano-beams and nano-plates [56,57,58].

**Figure 6:**
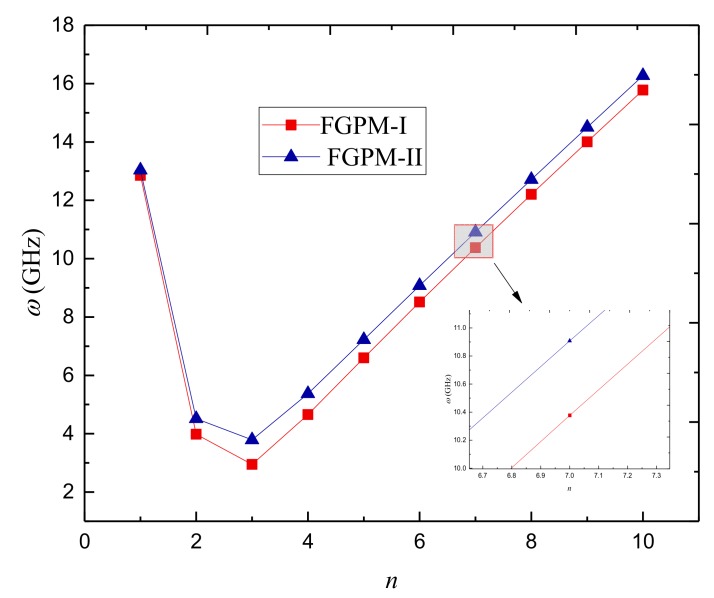
Variation of the natural frequency *ω*(GHz) against the circumferential wave number of different types of porous FGPM nanoshell.

In order to reveal the porosity type effect, the natural frequency against the circumferential wave number for FGPM-I and FGPM-II nanoshells is plotted in Figure 6. One can see that the natural frequency of the FGPM-II nanoshell is close to that of the FGPM-I one at small circumferential wave number. However, the natural frequency of the FGPM-II nanoshell becomes higher than that of its FGPM-I counterpart with the rise of circumferential wave number. The difference between them gets more and more obvious as the circumferential wave number increases further.

Table 12 gives the natural frequencies of FGPM-I and FGPM-II nanoshells for various porosity volume fractions. One can find that the larger nano-void volume fraction leads to the lower natural frequency of the FGPM-I nanoshell, while it leads to the higher natural frequency of the FGPM-II nanoshell. Therefore, it can be concluded that the porosity distribution type has a notable impact on vibration characteristics of FGPM nanoshells.

## 5. Conclusions

In this work, free vibration of porous FGPM nanoshells subjected to thermal and electrical loads is studied in the framework of Love’s shell theory and nonlocal elasticity theory. Size-dependent governing equations and boundary conditions are obtained based on Hamilton’s principle. Then, natural frequencies of the porous FGPM cylindrical nanoshells are obtained via the Navier method as well as the Galerkin method. The following conclusions were drawn:

(1) The fundamental natural frequency of the porous FGPM nanoshell decreases initially and then increases as the radius-to-thickness ratio increases. Furthermore, the fundamental frequency decreases with the rise of the length-to-radius ratio; especially, the frequency changes notably when the length-to-radius ratio is small;
(2) Applying positive voltage decreases the stiffness while applying negative voltage increases the stiffness of the porous FGPM cylindrical nanoshell. Furthermore, the temperature rise results in a reduction in the stiffness. In addition, the larger power-law index leads to the lower natural frequencies of the porous FGPM cylindrical nanoshell;
(3) The nonlocal parameter has a softening effect on the free vibrations of the porous FGPM nanoscale shells;
(4) The Galerkin solution procedure is an alternative method, which can give numerical results with satisfactory accuracy;
(5) Increasing the porosity volume fraction has a different effect on the natural frequencies of the FGPM-I and FGPM-II nanoshells, which shows that the porosity distribution type plays a notable role on vibration characteristics of the FGPM nanoscale shells.

## Figures and Tables

**Table 1:** Values of *c*_1_, *c*_2_, *c*_3_, *c*_4_, *ζ_i_* and *λ_i_* for different boundary conditions.

Boundary Condition	*c*_1_	*c*_2_	*c*_3_	*c*_4_	*ζ_i_*	*λ_i_*				
C-SS	1	−1	1	−1	$\frac{\cosh\left(\lambda_{i}\right)-\cos\left(\lambda_{i}\right)}{\sinh\left(\lambda_{i}\right)-\sin\left(\lambda_{i}\right)}$	3.9266	7.0686	10.2102	13.3518	…
C-C	1	−1	1	−1	$\frac{\cosh\left(\lambda_{i}\right)-\cos\left(\lambda_{i}\right)}{\sinh\left(\lambda_{i}\right)-\sin\left(\lambda_{i}\right)}$	4.7300	7.8532	10.9956	14.1372	…

**Table 2:** Comparison of natural frequency *ω*(GHz) of a SS-SS homogeneous piezoelectric nanoshell (*μ* = *e*_0_*a*/*L*).

*n*	*μ* = 0.02		*μ* = 0.04	
	Ke et al. [31]	Present	Ke et al. [31]	Present
1	0.4448	0.4448	0.4105	0.4105
2	0.2190	0.2190	0.1748	0.1748
3	0.4296	0.4296	0.3016	0.3016
4	0.7235	0.7235	0.4630	0.4630
5	1.0361	1.0361	0.6223	0.6223
6	1.3532	1.3532	0.7780	0.7780
7	1.6694	1.6694	0.9309	0.9309
8	1.9829	1.9829	1.0827	1.0827
9	2.2933	2.2933	1.2310	1.2310
10	2.6008	2.6008	1.3791	1.3791

**Table 3:** Comparison of natural frequency *ω*(GHz) of a C-SS homogeneous piezoelectric nanoshell (*μ* = *e*_0_*a*/*L*).

*n*	*μ* = 0.02		*μ* = 0.04	
	Ke et al. [31]	Present	Ke et al. [31]	Present
1	0.6189	0.6539	0.5710	0.6031
2	0.2701	0.2751	0.2155	0.2195
3	0.4357	0.4362	0.3058	0.3061
4	0.7247	0.7248	0.4637	0.4638
5	1.0365	1.0367	0.6225	0.6226
6	1.3534	1.3535	0.7781	0.7782
7	1.6695	1.6696	0.9309	0.9310
8	1.9830	1.9831	1.0817	1.0818
9	2.2934	2.2935	1.2310	1.2311
10	2.6008	2.6009	1.3791	1.3792

**Table 4:** Comparison of natural frequency *ω*(GHz) of a C–C homogeneous piezoelectric nanoshell (*μ* *= e*_0_*a*/*L*).

*n*	*μ* = 0.02		*μ* = 0.04	
	Ke et al. [31]	Present	Ke et al. [31]	Present
1	0.7987	0.8487	0.7368	0.7823
2	0.3386	0.3488	0.2702	0.2782
3	0.4458	0.4472	0.3129	0.3138
4	0.7266	0.7268	0.4649	0.4651
5	1.0371	1.0373	0.6228	0.6229
6	1.3536	1.3538	0.7782	0.7783
7	1.6696	1.6698	0.9310	0.9311
8	1.9830	1.9832	1.0818	1.0819
9	2.2934	2.2936	1.2310	1.2311
10	2.6008	2.6010	1.3791	1.3792

**Table 5:** Material properties of PZT-4 and PZT-5H [31,55].

Material	PZT-4	PZT-5H
Elastic constants (GPa)	*c*_11_ = 132, *c*_12_ = 71, *c*_13_ = 73, *c*_22_ = 132, *c*_23_ = 73, *c*_33_ = 115, *c*_66_ = 30.5	*c*_11_ = 126, *c*_12_ = 79.1, *c*_13_ = 83.9, *c*_22_ = 139, *c*_23_ = 83.9, *c*_33_ = 117, *c*_66_ = 23.5
Piezoelectric constants (C/m^2^)	*e*_31_ = −4.1, *e*_32_ = −4.1, *e*_33_ = 14.1	*e*_31_ = −6.5, *e*_32_ = −6.5, *e*_33_ = 23.3
Dielectric constants (10^−9^ C/Vm)	*s*_11_ = 5.841, *s*_33_ = 7.124	*s*_11_ = 15.05, *s*_33_ = 13.02
Thermal moduli (10^5^ N/km^2^)	*β*_11_ = 4.738, *β*_22_ = 4.738, *β*_33_ = 4.529	*β*_11_ = 4.738, *β*_22_ = 4.738, *β*_33_ = 4.529
Pyroelectric constant (10^−6^ C/N)	*p*_3_ = 25	*p*_3_ = 25
Mass density (kg/m^3^)	*ρ* = 7500	*ρ* = 7500

**Table 6:** Variation of the natural frequency *ω*(GHz) against the circumferential wave number *n* for different porosity volume fractions α of FGPM-I nanoshell (SS-SS).

*n*	*α* = 0	*α* = 0.1	*α* = 0.2
1	12.216	12.120	11.998
2	4.212	4.176	4.131
3	3.575	3.554	3.528
4	5.129	5.109	5.084
5	6.934	6.912	6.884
6	8.737	8.712	8.680
7	10.514	10.486	10.450
8	12.267	12.235	12.195
9	14.000	13.965	13.920
10	15.718	15.679	15.630

**Table 7:** Variation of the natural frequency *ω*(GHz) against circumferential wave number *n* for different porosity volume fractions α of FGPM-I nanoshell (C-SS).

*n*	*α* = 0	*α* = 0.1	*α* = 0.2
1	15.958	15.833	15.675
2	6.000	5.951	5.889
3	4.042	4.017	3.985
4	5.223	5.202	5.176
5	6.961	6.939	6.911
6	8.750	8.724	8.692
7	10.522	10.493	10.458
8	12.273	12.241	12.201
9	14.005	13.969	13.925
10	15.722	15.683	15.634

**Table 8:** Variation of the natural frequency *ω*(GHz) against circumferential wave number *n* for different porosity volume fractions α of FGPM-I nanoshell (C-C).

*n*	*α* = 0	*α* = 0.1	*α* = 0.2
1	18.371	18.228	18.048
2	7.670	7.609	7.531
3	4.657	4.626	4.587
4	5.365	5.343	5.314
5	7.000	6.977	6.948
6	8.763	8.738	8.706
7	10.529	10.500	10.464
8	12.277	12.245	12.205
9	14.008	13.972	13.928
10	15.724	15.685	15.636

**Table 9:** Variation of natural frequency *ω*(GHz) against the radius-to-thickness ratio *R/h* for different porosity volume fractions of FGPM-I nanoshell (*n* = 3, *L* = 300 *h*, *N* = 20).

*R/h*	*α* = 0	*α* = 0.1	*α* = 0.2
50	3.575	3.554	3.528
55	3.353	3.332	3.304
60	3.239	3.217	3.189
65	3.211	3.187	3.158
70	3.248	3.223	3.192
75	3.333	3.307	3.274
80	3.452	3.425	3.390
85	3.594	3.565	3.529
90	3.751	3.721	3.683
95	3.917	3.885	3.845
100	3.575	3.554	3.528

**Table 10:** Variation of the natural frequency *ω*(GHz) against circumferential wave number *n* for different power-law indexes *N* of FGPM-I nanoshell.

*n*	*N* = 0.3	*N* = 1	*N* = 5
1	13.474	12.852	12.305
2	4.437	3.982	3.967
3	3.422	2.950	3.088
4	5.027	4.656	4.735
5	6.929	6.602	6.630
6	8.822	8.512	8.503
7	10.680	10.376	10.336
8	12.508	12.203	12.135
9	14.312	14.002	13.908
10	16.097	15.779	15.661

**Table 11:** Variation of natural frequency *ω*(GHz) against the circumferential wave number *n* for different nonlocal parameter *e*_0_*a* of FGPM-I nanoshell.

*n*	*e*_0_*a* = 0	*e*_0_*a* = 1 nm	*e*_0_*a* = 1.5 nm	*e*_0_*a* = 2 nm
1	14.101	13.755	13.356	14.101
2	5.168	4.775	4.392	5.168
3	4.650	3.971	3.433	4.650
4	8.839	6.879	5.630	8.839
5	14.826	10.455	8.193	14.826
6	22.203	14.182	10.752	22.203
7	30.928	17.943	13.267	30.928
8	40.993	21.693	15.738	40.993
9	52.398	25.414	18.172	52.398
10	65.143	29.101	20.575	65.143

**Table 12:** Variation of natural frequency *ω*(GHz) against length-to-radius ratio *L*/*R* of different types of porous FGPM cylindrical nanoshell (*n* = 3, *N* = 20).

*L/R*	*α* = 0	*α* = 0.1		*α* = 0.2	
	Prefect	FGPM-I	FGPM-II	FGPM-I	FGPM-II
6	3.575	3.554	3.609	3.528	3.643
12	3.168	3.151	3.208	3.130	3.249
18	3.137	3.121	3.177	3.100	3.218
24	3.130	3.114	3.170	3.093	3.211
30	3.127	3.111	3.168	3.091	3.209
36	3.126	3.110	3.166	3.090	3.208
42	3.126	3.109	3.166	3.089	3.207
48	3.125	3.109	3.165	3.089	3.207

## Appendix A

(A1) $$\begin{array}{l} q_{11}=\frac{\left(-A_{66}n^{2}-A_{11}k_{m}{}^{2}R^{2}+a^{2}e_{0}{}^{2}I_{1}n^{2}\omega^{2}+I_{1}R^{2}\omega^{2}+a^{2}e_{0}{}^{2}I_{1}k_{m}{}^{2}R^{2}\omega^{2}\right)}{R^{2}}\\ q_{12}=\frac{\left(A_{12}k_{m}nR+A_{66}k_{m}nR\right)}{R^{2}}\\ q_{13}=\frac{\left(B_{12}k_{m}n^{2}+2B_{66}k_{m}n^{2}+A_{12}k_{m}R+B_{11}k_{m}{}^{3}R^{2}\right)}{R^{2}}\\ q_{14}=F_{31}k_{m}\\ q_{21}=-\frac{\left(-A_{12}k_{m}n-A_{66}k_{m}n-B_{12}k_{m}n-B_{66}k_{m}n\right)}{R}\\ q_{22}=-\frac{1}{R^{4}}(D_{22}n^{2}+B_{11}n^{2}R^{3}+D_{66}k_{m}{}^{2}R^{2}+A_{22}n^{2}R^{2}+B_{66}k_{m}{}^{2}R^{3}\\ \;+A_{66}k_{m}{}^{2}R^{4}-a^{2}e_{0}{}^{2}I_{1}n^{2}R^{2}\omega^{2}-I_{1}R^{4}\omega^{2}-a^{2}e_{0}{}^{2}I_{1}k_{m}{}^{2}R^{4}\omega^{2})\\ q_{23}=-\frac{\left(D_{22}n^{3}+B_{11}n^{3}R+A_{22}nR^{2}+D_{12}k_{m}{}^{2}nR^{2}+2D_{66}k_{m}{}^{2}nR^{2}+B_{12}k_{m}{}^{2}nR^{3}+2B_{66}k_{m}{}^{2}nR^{3}\right)}{R^{4}}\\ q_{24}=-\frac{\left(E_{32}nR^{2}+F_{31}nR^{3}\right)}{R^{4}}\\ q_{31}=-\frac{\left(-B_{12}k_{m}n^{2}-2B_{66}k_{m}n^{2}-A_{12}k_{m}R-B_{11}k_{m}{}^{3}R^{2}\right)}{R^{2}}\\ q_{32}=-\frac{1}{R^{4}}(D_{22}n^{3}+B_{11}n^{3}R+A_{22}nR^{2}+D_{12}k_{m}{}^{2}nR^{2}\\ \;+2D_{66}k_{m}{}^{2}nR^{2}+B_{12}k_{m}{}^{2}nR^{3}+2B_{66}k_{m}{}^{2}nR^{3})\\ q_{33}=-\frac{1}{R^{4}}(D_{22}n^{4}+B_{11}n^{2}R+A_{22}R^{2}+2D_{12}k_{m}{}^{2}n^{2}R^{2}+4D_{66}k_{m}{}^{2}n^{2}R^{2}-n^{2}N_{\theta 1}R^{2}\\ \;-a^{2}e_{0}{}^{2}k_{m}{}^{2}n^{2}N_{\theta 1}R^{2}+B_{12}k_{m}{}^{2}R^{3}+D_{11}k_{m}{}^{4}R^{4}-k_{m}{}^{2}N_{x1}R^{4}-a^{2}e_{0}{}^{2}k_{m}{}^{4}N_{x1}R^{4}\\ \;-a^{2}e_{0}{}^{2}I_{1}n^{2}R^{2}\omega^{2}-I_{1}R^{4}\omega^{2}-a^{2}e_{0}{}^{2}I_{1}k_{m}{}^{2}R^{4}\omega^{2})\\ q_{34}=-\frac{\left(E_{32}n^{2}+F_{31}R+E_{31}k_{m}{}^{2}R^{2}\right)}{R^{2}}\\ q_{41}=-F_{31}k_{m}\\ q_{42}=-\frac{\left(-E_{32}n-F_{31}nR\right)}{R^{2}}\\ q_{43}=-\frac{\left(-E_{32}n^{2}-E_{31}k_{m}{}^{2}R^{2}\right)}{R^{2}}\\ q_{44}=-\left(k_{m}{}^{2}X_{11}+n^{2}X_{22}+X_{33}\right) \end{array}$$

where $k_{m}=m\pi /L$.
